# Switchable Altermagnetism in a Layered van der Waals Metal–Organic Framework Driven by Spin-Crossover

**DOI:** 10.1021/jacs.6c15502

**Published:** 2026-09-23

**Authors:** Diego López-Alcalá, Alberto M. Ruiz, Andrei Shumilin, José J. Baldoví

**Affiliations:** † Instituto de Ciencia Molecular, 16781 Universitat de València, Catedrático José Beltrán 2, 46980 Paterna, Spain

## Abstract

Dynamical control of altermagnetism is a key requirement for translating its unique spin-dependent functionalities into practical spintronic devices, yet effective switching mechanisms remain largely unexplored. Here, we open an unprecedented, versatile, and programmable route based on spin-crossover to switch altermagnetism on demand in molecular materials. Using density functional theory calculations, we demonstrate an altermagnetic ground state in the layered van der Waals metal–organic frameworks MnX_2_(tdz)_2_ (X = Cl, Br; tdz = thiadiazole), stabilized by anisotropic interlayer exchange interactions, which gives rise to a characteristic d-wave momentum-space spin splitting and an associated spin-splitter transport response. Our findings reveal that under hydrostatic pressure, a high-spin-to-low-spin transition reconfigures the Mn d-orbital occupation, thus modifying the magnetic exchange network and stabilizing a different antiferromagnetic ground state whose symmetry suppresses the nonrelativistic spin splitting. Crucially, spin-crossover switches altermagnetism not by directly altering the electronic structure but by changing the symmetry of the magnetic ground state. These results establish molecular spin-crossover altermagnets as a platform for externally reconfigurable spintronic devices.

## Graphical abstract

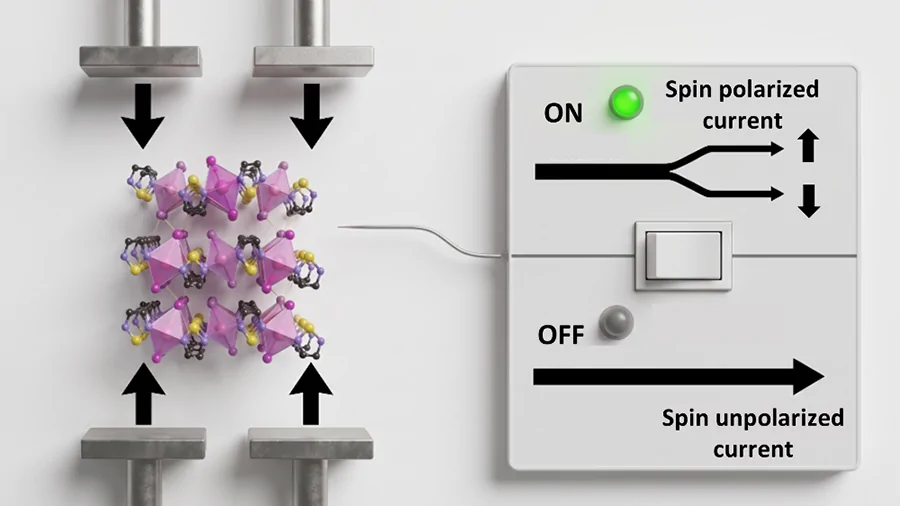

Altermagnetism has recently emerged as an unconventional magnetic phase in which collinear antiferromagnets exhibit sizable nonrelativistic spin splitting while preserving zero net magnetization.^1,2^ Governed by crystal symmetry, this spin splitting enables spin-dependent transport and current-induced spin functionalities without relying on spin–orbit coupling (SOC).^3^ While numerous altermagnetic (AM) materials have recently been identified, achieving dynamic control over their magnetic symmetry and spin splitting remains a major challenge toward practical spintronic applications.^4^

Metal–organic frameworks (MOFs) emerge as an attractive platform to address this challenge due to their exceptional chemical versatility and modular nature, which enable the rational engineering of crystal symmetry, magnetic exchange interactions, and electronic structure.^5–7^ Moreover, MOFs exhibit a broad range of electronic and magnetic functionalities, including room-temperature magnetism, topological magnetic phases, and chemically tunable magnetic interactions.^8–13^ Within this context, coordination networks have recently been proposed as promising AM materials.^14^ These span from architectures at the 2D limit to bulk systems.^15–20^ However, while these studies considerably expand the family of altermagnets through chemical design, the resulting magnetic properties remain essentially static, precluding dynamic manipulation of the magnetic symmetry and associated spin splitting.

Among the most distinctive functionalities of magnetic molecular materials is spin-crossover (SCO), which enables reversible changes in the electronic configuration and magnetic state under external stimuli such as pressure, temperature, or light.^21,22^ Coupling SCO with altermagnetism therefore represents a promising strategy to dynamically manipulate magnetic symmetry and switch nonrelativistic spin splitting on demand.^23,24^ Nevertheless, no molecular material has yet been shown to intrinsically couple SCO and altermagnetism, leaving switchable AM functionalities in molecular materials unrealized.

In this work, we demonstrate that SCO provides an effective strategy for switching altermagnetism in molecular materials. We investigate the layered van der Waals MOFs MnX_2_(tdz)_2_ (X = Cl, Br; tdz = thiadiazole) by using density functional theory (DFT) calculations. Our simulations reveal an AM ground state stabilized by anisotropic interlayer exchange interactions, which give rise to a characteristic d-wave spin splitting along the stacking direction. Then, we apply hydrostatic pressure, and, interestingly, an SCO transition that reconfigures the Mn d-orbital occupation takes place, which changes the magnetic ground state, accompanied by a transition from an AM to a conventional antiferromagnetic (AFM) phase.

The chemical structure of our case study, i.e., MnX_2_(tdz)_2_, belongs to the orthorhombic *Cmce* (No. 64) space group, in which the Mn^2+^ centers adopt slightly distorted octahedral environments (Figure 1). These are coordinated by axial halide ligands and equatorial tdz molecules. The alternating orientation of neighboring tdz ligands generates two crystallographically inequivalent Mn sublattices while preserving the global crystal symmetry. This provides the structural framework required for the symmetry-protected altermagnetism. This layered topology closely resembles that of the experimentally synthesized NCS analogue,^25^ which supports its structural feasibility, and periodic DFT studies of Mn­(II) MOFs have previously addressed their electronic structure and magnetic exchange interactions.^26^ Furthermore, ab initio molecular dynamics (AIMD) simulations confirm the dynamical stability of both MnCl_2_(tdz)_2_ and MnBr_2_(tdz)_2_, without structural reconstruction (Figures S6 and S7).

**Figure 1:**
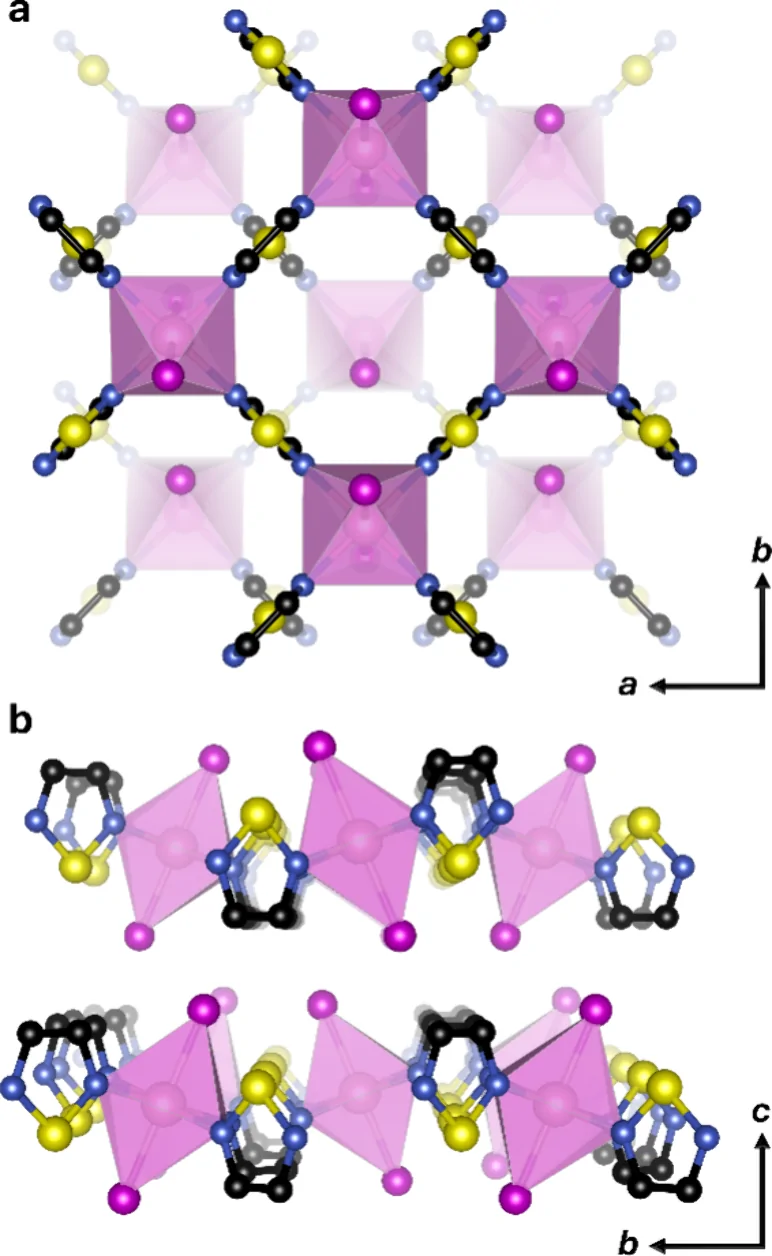
(a) Top and (b) side views of MnCl_2_(tdz)_2_ structure. Color code: pink, Mn; purple, Cl; yellow, S; blue, N; black, C. H atoms have been omitted for clarity.

The Mn^2+^ nodes display a calculated magnetic moment of 4.52 μ_B_, which is consistent with a high-spin (HS) d^5^ magnetic configuration (t_2g_^3^e_g_^2^, *S* = ^5^/_2_). Superexchange mediated by the tdz ligands gives rise to several competing magnetic configurations (Figure 2), whose distinct symmetry relationships determine whether altermagnetism is allowed.

**Figure 2:**
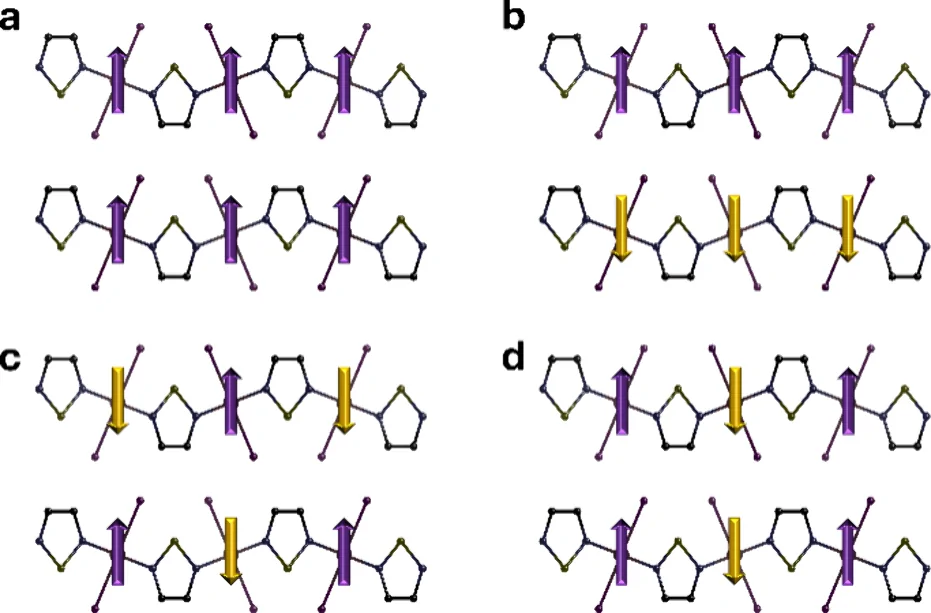
Spin orientations of (a) FM, (b) AFM_1_, (c) AFM_2_, and (d) AFM_3_ configurations. Color code: purple (orange) represents spin up (down).

The symmetry relationship between opposite-spin sublattices is decisive for altermagnetism. On one hand, in AFM_1_ and AFM_2_ configurations the [C_2_∥*i*] symmetry operation connects opposite-spin sublattices by inversion and therefore forbids altermagnetism. Conversely, inAFM_3_ those are related by glide operations, fulfilling the symmetry conditions required for an AM ground state (see extended discussion in Supplementary Note 1).^2^

Our calculations identify AFM_3_ as the lowest-energy magnetic configuration for both MnCl_2_(tdz)_2_ and MnBr_2_(tdz)_2_ (Table 1), demonstrating that the energetically preferred magnetic state is precisely the one compatible with altermagnetism. The stabilization of AFM_3_ originates from the dominant AFM superexchange expected for HS Mn^2+^, where the partially occupied t_2g_ and e_g_ orbitals strongly favor AFM coupling over the ferromagnetic alignment. Therefore, the existence of altermagnetism in MnX_2_(tdz)_2_ relies on the stability of the AFM_3_ spin arrangement. Numerical errors in relative magnetic energies are below 0.006 meV/Mn (Table S1).

**Table 1:** Calculated Energy of Each Magnetic Configuration in MnX_2_(tdz)_2_ in meV/Mn

	DFT	FM	AFM_1_	AFM_2_	AFM_3_
MnCl_2_(tdz)_2_	PBE+U	5.61	4.47	0.77	0
	HSE06	4.48	4.03	0.42	0
MnBr_2_(tdz)_2_	PBE+U	4.86	3.44	1.02	0
	HSE06	3.80	3.16	0.62	0

Having established AFM_3_ as the symmetry-allowed magnetic ground state, we next examined its electronic signature (Figures 3a and S8). MnCl_2_(tdz)_2_ is a semiconductor with a direct band gap of 1.52 eV [1.66 eV in MnBr_2_(tdz)_2_]. As the projected density of states (PDOS) reveals (Figure 3b), the valence-band maximum is dominated by localized Mn d states, whereas the conduction-band minimum mainly originates from tdz orbitals, indicating a metal-to-ligand charge-transfer gap.^27,28^

**Figure 3:**
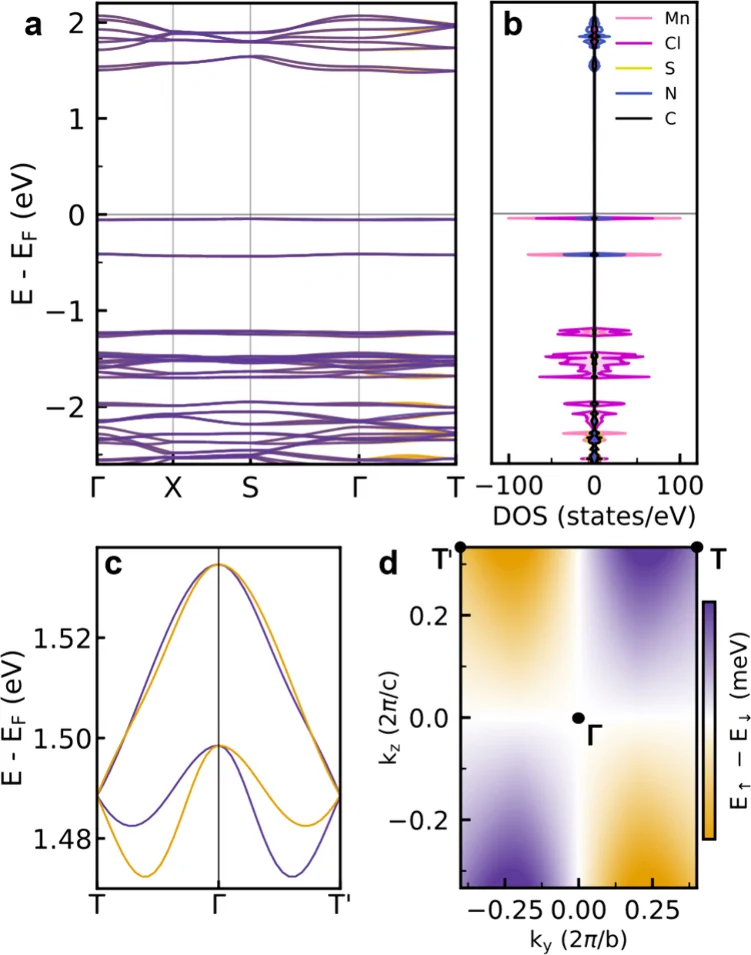
(a) Electronic band structure and (b) PDOS in MnCl_2_(tdz)_2_. (c) Spin splitting along the *b*∥*c* direction. (d) Full spin splitting along the *bc* plane.

Consistent with the symmetry of the AFM_3_ ground state, the electronic structure exhibits a highly anisotropic spin splitting in momentum space. While the electronic bands remain strictly spin degenerate along the in-plane crystallographic directions, finite spin splitting emerges exclusively along the stacking direction, allowing the characteristic alternating T−Γ–T′ dispersion expected for an altermagnet (Figure 3c). The corresponding spin-splitting map further reveals the characteristic d-wave anisotropy within the *bc* plane, with two nodal directions separating regions of opposite-spin polarization (Figure 3d).^1^ Our hybrid functional calculations further confirm that the observed spin splitting is an intrinsic electronic property of MnX_2_(tdz)_2_ rather than a functional-dependent artifact (Figure S10).

The persistence of this spin splitting upon inclusion of SOC confirms its purely nonrelativistic origin (Figure S11). The Br analogue exhibits the same electronic behavior despite the larger interlayer separation and enhanced relativistic effects associated with the heavier Br atoms, demonstrating the robustness of the AM ground state. More importantly, the pronounced momentum-space anisotropy of the spin splitting is expected to directly determine the spin-transport response of MnX_2_(tdz)_2_.

The strong directional dependence of the AM spin splitting naturally gives rise to an anisotropic spin-transport response.^29,30^ In particular, the symmetry relating inequivalent spin sublattices confines the AM signature to the *bc* plane, enabling a spin-splitter response in which spin-conserving transport deflects spin-up and spin-down carriers into opposite transverse channels. To verify this prediction, we perform Boltzmann transport calculations, revealing spin-dependent conductivity exclusively within the *bc* plane while remaining absent along the other crystallographic directions (Figures S12 and S13). This generates a spin accumulation on the opposite sides of the *bc* plane (Figure S4). The resulting spin-splitter response therefore constitutes the transport fingerprint of the anisotropic AM electronic structure in MnX_2_(tdz)_2_.

The anisotropic electronic and transport properties in MnX_2_(tdz)_2_ ultimately rely on the stability of the AFM_3_ ground state. To understand its microscopic origin, we evaluated the magnetic exchange interactions (*J*) governing the spin Hamiltonian (Supplementary Note 2). Three exchange pathways were considered, corresponding to the first-neighbor intralayer interaction (*J*_1_) and the first- and second-neighbor interlayer couplings (*J*_2_ and *J*_3_, respectively).

As summarized in Table 2, all exchange interactions are AFM, consistent with the dominant superexchange expected between HS Mn^2+^ centers. The AFM_3_ ground state is primarily stabilized by the cooperative action of *J*_1_ and *J*_2_, whereas the weaker AFM *J*_3_ couples second-neighbor spins that are ferromagnetically aligned, introducing a minor magnetic frustration that does not overcome the dominant exchange network.

**Table 2:** Calculated Magnetic Exchange Interactions (*J*, in meV) and Magnetic Anisotropy Energy (*D*, in meV/Mn)

	*J*_1_	*J*_2_	*J*_3_	*D*
MnCl_2_(tdz)_2_	–0.89	–0.77	–0.39	0.31
MnBr_2_(tdz)_2_	–0.69	–0.61	–0.31	0.70

Both compounds exhibit in-plane magnetic anisotropy, with energies comparable to those of other layered van der Waals magnets.^31^ As expected, magnetic anisotropy increases in MnBr_2_(tdz)_2_ due to the stronger SOC associated with Br atoms. Atomistic spin dynamics simulations predict Néel temperatures (*T*_N_) of approximately 30 K (25 K) for MnCl_2_(tdz)_2_ (MnBr_2_(tdz)_2_) (Figure S14), in good agreement with experimentally reported values for related layered magnetic MOFs.^32,33^ Together, these results demonstrate that the AFM_3_ ground state is magnetically robust, providing the stability required for the AM electronic structure and spin-splitter response described above.

Having established the microscopic origin of the AM ground state, we now investigate whether it can be dynamically controlled by external stimuli. We apply hydrostatic pressure up to 10 GPa to MnX_2_(tdz)_2_. Our results reveal a sharp HS-to-low-spin (LS) transition above ∼5 GPa. As shown in Figure 4a, the Mn magnetic moment abruptly decreases from ∼4.5 to ∼1 μ_B_, providing clear evidence of SCO. This transition is accompanied by a pronounced collapse of the unit-cell volume (Figure 4b), consistent with the reduced ionic radius of LS Mn^2+^. Similar pressure-induced SCO transitions have been reported in octahedrally coordinated Mn compounds, although typically at considerably higher pressures (≈20–30 GPa).^34–37^ The substantially lower transition pressure predicted here is attributed to the structural flexibility of the organic framework, which facilitates the electronic reconfiguration.^38,39^

**Figure 4:**
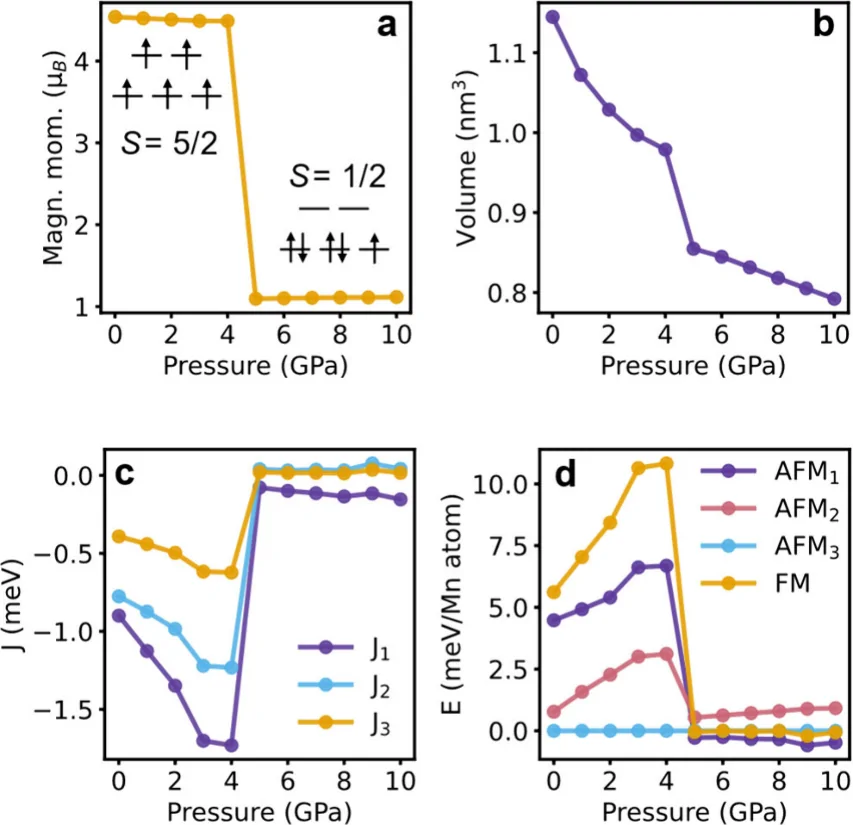
Effect of hydrostatic pressure on the (a) magnetic moment in Mn atoms, (b) unit-cell volume, (c) magnetic exchange couplings, and (d) energy of each magnetic configuration in MnCl_2_(tdz)_2_.

The pressure-induced SCO transition reconfigures the Mn d-orbital occupation, modifying the magnetic exchange interactions (Figure 4c). More broadly, pressure can alter molecular magnetic functionality through changes in the local coordination environment, crystal-field anisotropy, and molecular vibrations, as demonstrated in single-molecule magnets.^40,41^ Before the transition, hydrostatic pressure strengthens the dominant AFM exchange interactions (*J*_1_ and *J*_2_), reinforcing the stability of the AFM_3_ ground state and raising *T*_N_ to approximately 55 K (Figure S15). Thus, the nonrelativistic spin splitting is enhanced by nearly 100%, together with the associated spin-splitter response (Figures S16–S18). Therefore, moderate pressure does not merely preserve the AM phase but also actively reinforces its electronic and transport functionalities.

Beyond the SCO, the pressure response changed qualitatively. The depopulation of the Mn e_g_ orbitals suppresses the dominant superexchange pathways present in the HS configuration, leaving only weaker t_2g_-mediated interactions.^42^ Consequently, all *J* values decrease abruptly. More importantly, the SCO stabilizes a different magnetic ground state: AFM_1_ becomes energetically preferred over AFM_3_ (Figure 4d). Unlike AFM_3_, the AFM_1_ configuration preserves the [*C*_2_∥*i*] symmetry operation relating opposite-spin sublattices, thereby restoring the symmetry that forbids altermagnetism. Accordingly, the electronic band structure becomes fully spin degenerate above the transition pressure, and the spin-splitter response disappears (Figure S19).

These results demonstrate that the cancellation of spin splitting is not a direct consequence of hydrostatic pressure itself but rather of the pressure-induced SCO transition that changes the symmetry of the magnetic ground state. Because SCO can also be triggered by temperature, light, or other external stimuli in molecular materials, with oriented external electric fields offering a route to directly tune the spin-transition energetics,^43^ this mechanism extends well beyond pressure control, providing a versatile approach to designing switchable altermagnetic materials. Rather than simply modifying magnetic interactions, SCO changes the symmetry of the magnetic ground state, thereby switching the AM functionality.

In summary, we demonstrate that SCO provides an effective mechanism for dynamically switching altermagnetism in molecular materials. Using the layered van der Waals MOFs MnX_2_(tdz)_2_ as a proof of concept, we show that anisotropic interlayer exchange stabilizes an AM ground state characterized by d-wave momentum-space spin splitting and a corresponding spin-splitter transport response. Under hydrostatic pressure, a HS-to-LS transition reconfigures the Mn d-orbital occupation, modifies the magnetic exchange network, and stabilizes a different AFM ground state whose symmetry suppresses the nonrelativistic spin splitting.

These results demonstrate that the disappearance of altermagnetism is not a direct consequence of pressure itself but rather of the SCO-driven change in magnetic symmetry. The reversibility of pressure-induced SCO has been demonstrated in 2D coordination polymers, where pressure cycling recovers the initial spin state and gives rise to piezohysteresis.^44,45^ More broadly, this work establishes molecular altermagnets exhibiting SCO as a versatile platform for achieving dynamically switchable altermagnetism. Our results introduce a design principle for externally controllable spin-dependent functionalities and pave the way for adaptive programmable molecular spintronic devices.

## Computational Details

Structural relaxations, electronic structure calculations, magnetic configurations and AIMD simulations were performed using the Vienna Ab initio Simulation Package.^46^ The exchange-correlation energy was described within the generalized gradient approximation (GGA) using the Perdew–Burke–Ernzerhof functional. Grimme’s D3 dispersion correction was included to account for the weak van der Waals interactions between adjacent MOF layers. The partially occupied Mn 3d orbitals were treated within the GGA+U approach using an effective Hubbard parameter *U*_eff_ = *U* – *J* = 3 eV. Hybrid HSE06 calculations were additionally performed as a computational validation of the relative stability of the magnetic configurations and the predicted AM spin splitting. The projector-augmented-wave method was employed with a plane-wave energy cutoff of 500 eV. Brillouin-zone integrations were carried out using a Monkhorst–Pack *k*-point mesh of 5 × 5 × 3. The electronic self-consistency criterion was set to 10^–7^ eV, and all structures were fully relaxed until the residual forces on each atom were below 0.01 eV Å^–1^. AIMD simulations were carried out within the canonical (*NVT*) ensemble for 5 ps using a time step of 1 fs at 300 and 600 K. Hydrostatic pressure was simulated as an energy correction to the stress tensor. Néel temperatures were evaluated via atomistic spin simulations, as implemented in Vampire software.^47^ A sample of 50 nm × 50 nm × 50 nm was used in combination with 10000 equilibration and loop time steps. Maximally localized Wannier functions were constructed using Wannier90 code,^48^ considering the d orbitals of Mn, the p orbitals of C, N, Cl, and S, and the s orbitals of H atoms as the basis set. Transport calculations were performed within Boltzmann formalism, as implemented in BoltzWann code.^49^ The scattering rate was set to Γ = 1 meV and the temperature to 20 K.

## Supplementary Material



## Notes

Symmetry analysis, spin Hamiltonian, spin accumulation due to spin-splitter effect, pressure-induced SCO, additional information (PDF)
