# Supplementary material for: Switchable Altermagnetism in a Layered van der Waals Metal–Organic Framework Driven by Spin-Crossover
Source: J Am Chem Soc. 2026 Sep 23;148(43):46500–5. doi: 10.1021/jacs.6c15502 (PMC13741834; doi:10.1021/jacs.6c15502)
Supplement: Supplementary file 1 [file ja6c15502_si_001.pdf]

Supporting Information

Switchable Altermagnetism in a Layered van der Waals Metal-Organic Framework Driven by Spin-Crossover

Diego López-Alcalá[1], Alberto M. Ruiz[1], Andrei Shumilin[1] and José J. Baldoví[1,*]

[1]Instituto de Ciencia Molecular, Universitat de València, Catedrático José Beltrán 2, 46980 Paterna, Spain

Supplementary Note 1: Symmetry analysis

The *Cmce* (64) space group of $MnX_2(tdz)_2$ MOFs contains an inversion center, shown in Figure S1a. In the AFM_1 and AFM_2 magnetic configurations, it gives rise to the $[C_2 \parallel i]$ symmetry, which prohibits altermagnetism. In contrast, the AFM_3 configuration is reduced to the $[1 \parallel i]$ symmetry. Nevertheless, the MOFs in this state preserve the $[C_2 \parallel g_y]$ and $[C_2 \parallel g_z]$ symmetries, where g_y and g_z denote the glide planes shown in Figure S1(b,c). Together, these symmetries result in a *d*-wave AM ground state, giving rise to the band spin splitting shown in Figure 3(c,d) of the main text and enabling spin-dependent conductivity in the yz-plane.

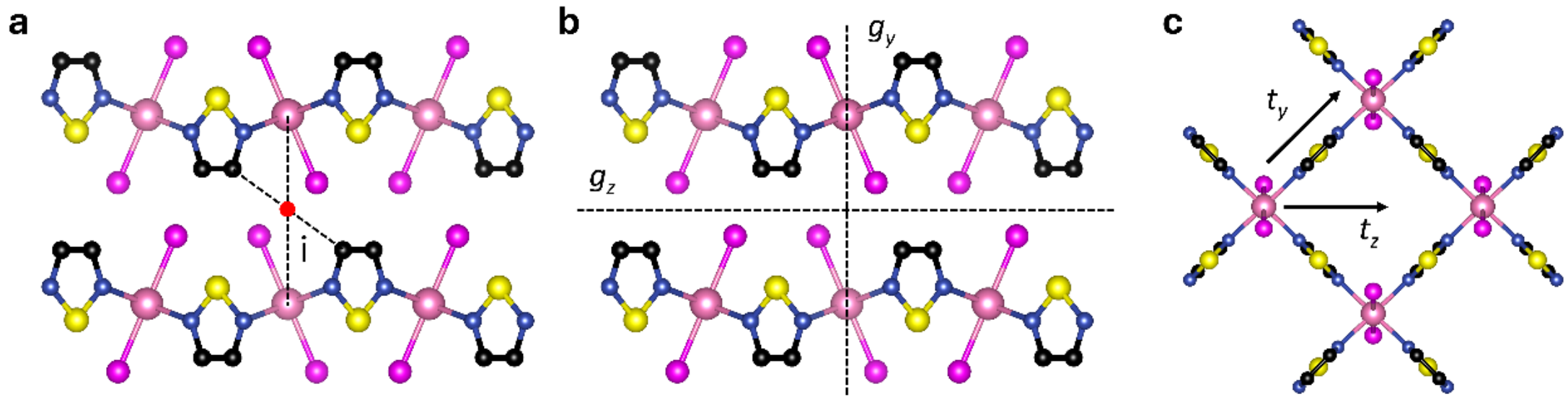

Figure S1. Key symmetry elements of $MnX_2(tdz)_2$. **a** Inversion center i. **b** Glide planes g_y and g_z. **c** Translation vectors t_y and t_z associated with the glide planes g_y and g_z respectively.

Supplementary Note 2: Spin Hamiltonian

The magnetic exchange interactions were estimated using the energy-difference approach within the framework of the Heisenberg spin Hamiltonian:

$$H = -\sum_{i \neq j} J_{ij} \vec{S_i} \cdot \vec{S_j} - \sum_{i} D_i (S_i)^2 \qquad (1)$$

where J_{ij} represents the magnetic interaction between two magnetic moments along the lattice (S_i and S_j) and D is the uniaxial magnetic anisotropy. Here, a positive value of J favors FM coupling and negative J value favors AFM coupling. Three inequivalent magnetic exchange pathways were considered, corresponding to the J_1, J_2 and J_3 interactions (Figure S2). Three antiferromagnetic spin configurations (AFM_A, AFM_B, and AFM_C in Figure S3)) were constructed to predominantly probe each of these exchange pathways, together with the ferromagnetic (FM) configuration. In all calculations, the crystal

structure and all computational parameters, including the exchange-correlation functional, Hubbard U, plane-wave cutoff energy, and k-point sampling, were kept identical. Only the initial spin arrangement of the Mn ions was modified.

The magnetic exchange constants were estimated from the total-energy differences between the FM and the corresponding AFM configurations according to

$$J_i = \frac{E_{\mathrm{FM}} - E_{\mathrm{AFM},i}}{8S^2} \quad (2)$$

where E_F and E_{AFMi} are the total energies of the ferromagnetic and the corresponding antiferromagnetic configurations, respectively, and S = 5/2 is the spin quantum number of the HS Mn^{2+} ions.

Accordingly, the three exchange parameters were obtained as

$$J_1 = \frac{E_{\mathrm{FM}} - E_{\mathrm{AFM}_A}}{8S^2} \quad (3)$$

$$J_2 = \frac{E_{\mathrm{FM}} - E_{\mathrm{AFM}_\mathrm{B}}}{8S^2} \quad (4)$$

$$J_3 = \frac{E_{\mathrm{FM}} - E_{\mathrm{AFM}_\mathrm{C}}}{8S^2} \quad (5)$$

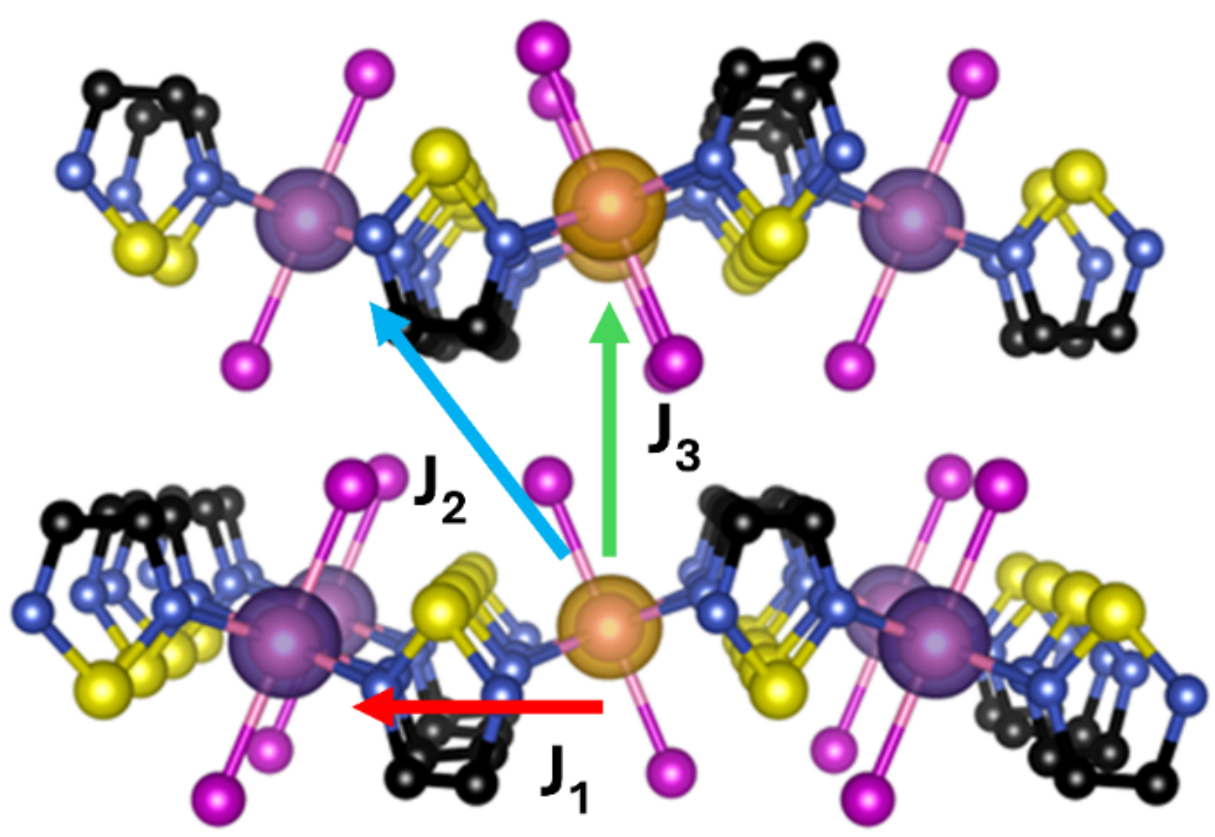

Figure S2. Schematic representation of the magnetic exchange interactions considered in the spin Hamiltonian.

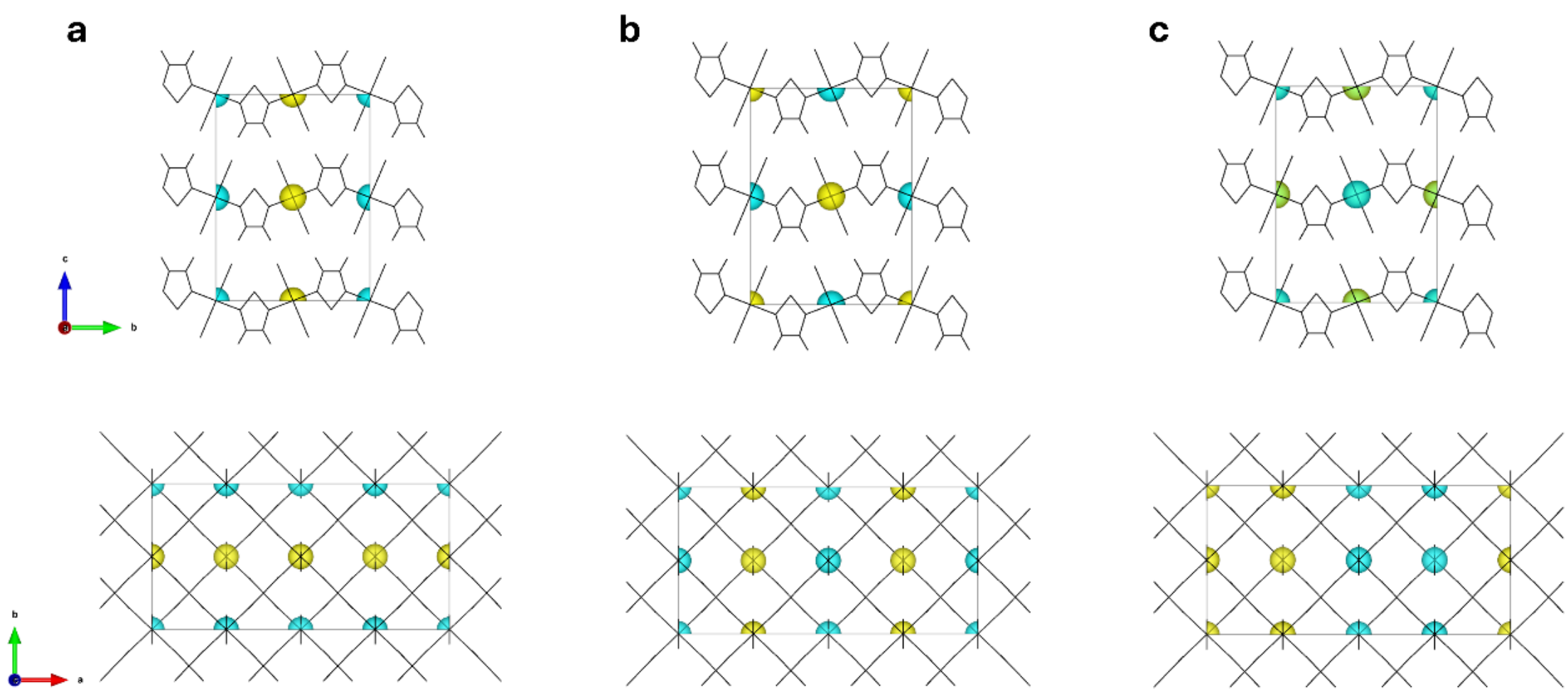

Figure S3. Spin alignment in AFM configurations used to estimate J. **a** AFM_A, **b** AFM_B and **c** AFM_C. Color code: yellow (blue) represents spin up (down).

Supplementary Note 3: Spin accumulation due to spin-splitter effect

To analyze the spin accumulation at the edges of a macroscopic sample, we map the Boltzmann transport theory onto spin-resolved diffusion equations,[1] assuming that, on sufficiently large length scales, momentum-relaxation processes maintain the electron distribution close to the Fermi distribution, with a position- and spin-dependent chemical potential μ. We further assume a single phenomenological spin relaxation time $\tau_s \gg \tau$, much longer than the momentum relaxation time τ. The applied electric field E and the resulting charge current are taken to be uniform and directed along the y-axis, while the spin current flows along the z-direction. Under these assumptions, the spin accumulation is given by

$$s(z) = \frac{El_s}{e} \frac{\sigma_{zy}^{\uparrow} - \sigma_{zy}^{\downarrow}}{\widetilde{D}\cosh(L/2l_s)} \sinh\left(\frac{z}{l_s}\right) \quad (6)$$

where the spin accumulation is defined as $s(z) = n_\uparrow - n_\downarrow$, with $n_\uparrow$ and $n_\downarrow$ denoting the densities of spin-up and spin-down electrons, respectively. e is the elementary charge, $\sigma_{zy}^{\uparrow}$ and $\sigma_{zy}^{\downarrow}$ are the off-diagonal components of the conductivity tensor for spin-up and spin-down electrons, respectively, and $\widetilde{D}$ is the effective spin diffusion constant, $\widetilde{D} = 2D_{yy}^{\uparrow}D_{yy}^{\downarrow}/(D_{yy}^{\uparrow}+D_{yy}^{\downarrow})$. The components of the diffusion tensor $D_{\alpha\beta}^{\uparrow,\downarrow}$ for the two spin channels are calculated from the Boltzmann formalism:

$$D_{\alpha\beta}^{\uparrow\downarrow} = \frac{\tau}{g} \sum_{\boldsymbol{k},b} v_{\alpha}^{(\boldsymbol{k},b,\uparrow\downarrow)} v_{\beta}^{(\boldsymbol{k},b,\uparrow\downarrow)} \frac{\partial f(\varepsilon_{\boldsymbol{k},b,\uparrow\downarrow})}{\partial \mu} \tag{7}$$

Here the summation runs over all wave vectors $\boldsymbol{k}$ and bands b, $v_{\alpha}^{(\mathbf{k},b,\uparrow,\downarrow)}$ is the α-component of the corresponding group velocity, $\varepsilon_{\mathbf{k},b,\uparrow,\downarrow}$ is the band energy, and $\partial f(\varepsilon)/\partial\mu$ is the derivative of the Fermi–Dirac distribution with respect to the chemical potential. The quantity g denotes the effective density of states.

$$g = \sum_{\boldsymbol{k},b} \frac{\partial f(\varepsilon_{\boldsymbol{k},b,\uparrow\downarrow})}{\partial \mu} \tag{8}$$

$l_s = \sqrt{\widetilde{D}\,\tau_s}$ is the spin relaxation length. *L* is the sample width; it is presumed that sample is described by $-\frac{L}{2} < z < \frac{L}{2}$.

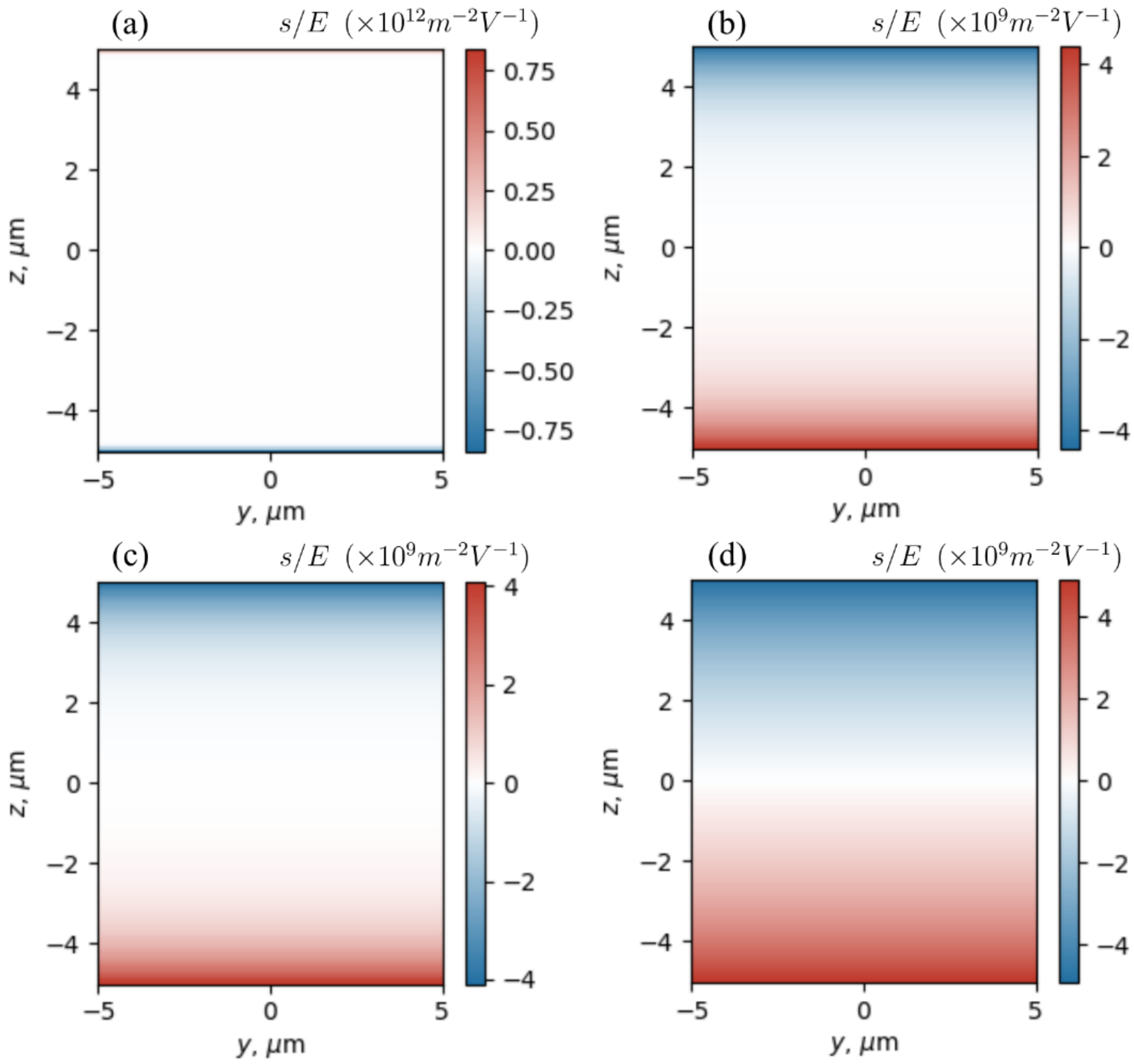

Figure S4. Simulated spin accumulation per unit electric field in $MnCl_2(tdz)_2$, with $\tau = 330\ fs$, $\tau_s = 10\ ns$ and $T = 20$ K in a *10* μm – wide sample. (a,b,c) correspond to *P* = 0 and different positions of the Fermi level, (a): $E_F = 0$, (b) $E_F = -0.85$ eV, (c) $E_F = 1.9$ eV. (d) spin accupulation per unit electric field at *P* = 4 GPa and $E_F = 0$.

In Figure S4 we demonstrate the spin acumulation calculated in $MnCl_2(tdz)_2$ for three positions of the chemical potential and with pressure P = 4 GPa and $E_F = 0$. It shows that a significant spin accumulation requires positioning of the chemical potential in a band with significant density of states and spin-splitting effect.

The transport results should be interpreted as a theoretical assessment of the spin selectivity associated with the AM electronic structure, rather than as evidence of an intrinsically conducting material. In its neutral form, $MnX_2(tdz)_2$ is a semiconductor, with a calculated band gap of approximately 1.52 eV for the Cl derivative. Consequently, observing the predicted spin-dependent transport experimentally would require introducing carriers so that the chemical potential reaches electronic states available for conduction. This could potentially be achieved by methods such as chemical doping, electrostatic gating, or interface-based charge transfer, approaches that have been explored to modify the electronic properties of MOFs.[2] Thus, the present results identify the spin-dependent transport response expected upon carrier injection and motivate the development of conducting or doped altermagnetic MOFs.

Supplementary Note 4: Pressure-induced SCO

To characterize the pressure-induced SCO, structural relaxations were performed at increasing hydrostatic pressures, using the converged HS solution at the preceding pressure as the initial magnetic configuration. From 0 to 4 GPa, the system consistently converges to the HS state, with the Mn magnetic moments and unit-cell volume evolving continuously with pressure. At 5 GPa, however, the structural relaxation undergoes an abrupt volume collapse accompanied by a pronounced reduction of the Mn magnetic moment, indicating a transition from the HS to a LS configuration.

The close relationship between the structural and spin-state changes is important for interpreting the transition. The HS and LS states are associated with substantially different coordination geometries and unit-cell volumes. Consequently, the LS electronic solution is not readily stabilized while the structure retains its HS-like volume and geometry. Indeed, fixed-geometry single-point calculations on the HS-relaxed structure can yield a large energy penalty for the LS solution, but this quantity does not represent the thermodynamic HS–LS energy difference because it does not account for the structural relaxation accompanying the SCO.

This behavior is consistent with the strong coupling between spin state and structural volume observed in pressure-induced SCO systems, where the reduction in molecular volume upon conversion to the LS state contributes to pressure-driven stabilization of the LS configuration, as observed in Mn(II)

honeycomb lattices.[3] In our system, the transition is therefore identified from the coupled discontinuities in unit-cell volume and Mn magnetic moment that emerge during pressure-driven structural relaxation, rather than from fixed-geometry HS–LS energy differences. Therefore, the abrupt changes at 5 GPa provide a direct signature of the pressure-induced SCO and define the transition pressure adopted throughout the manuscript.

Supplementary Note 5: Additional information

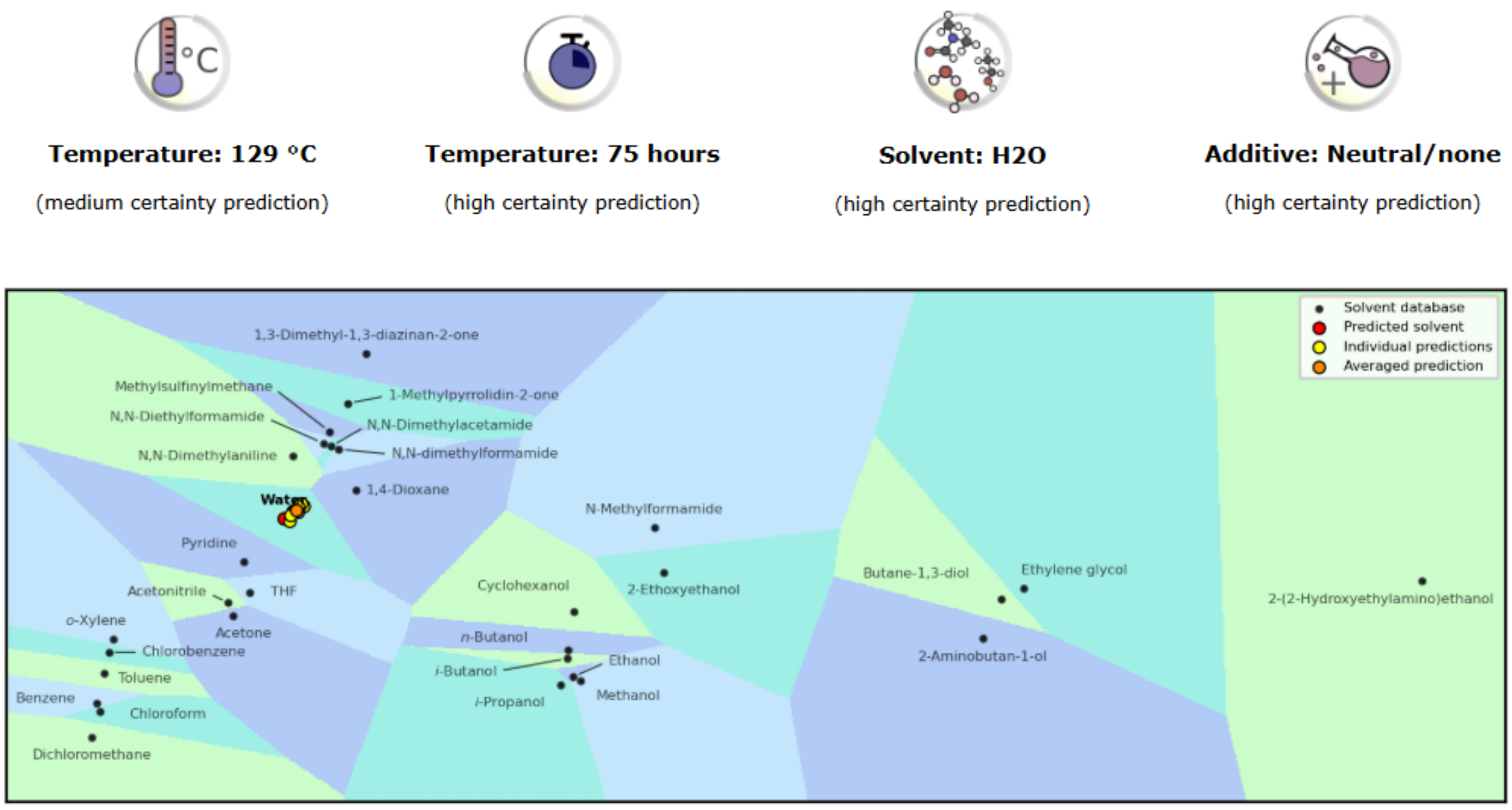

Figure S5. Synthesis protocol prediction of $MnCl_2(tdz)_2$. Obtained using machine learning tool available at https://mof-synthesis.aimat.science.

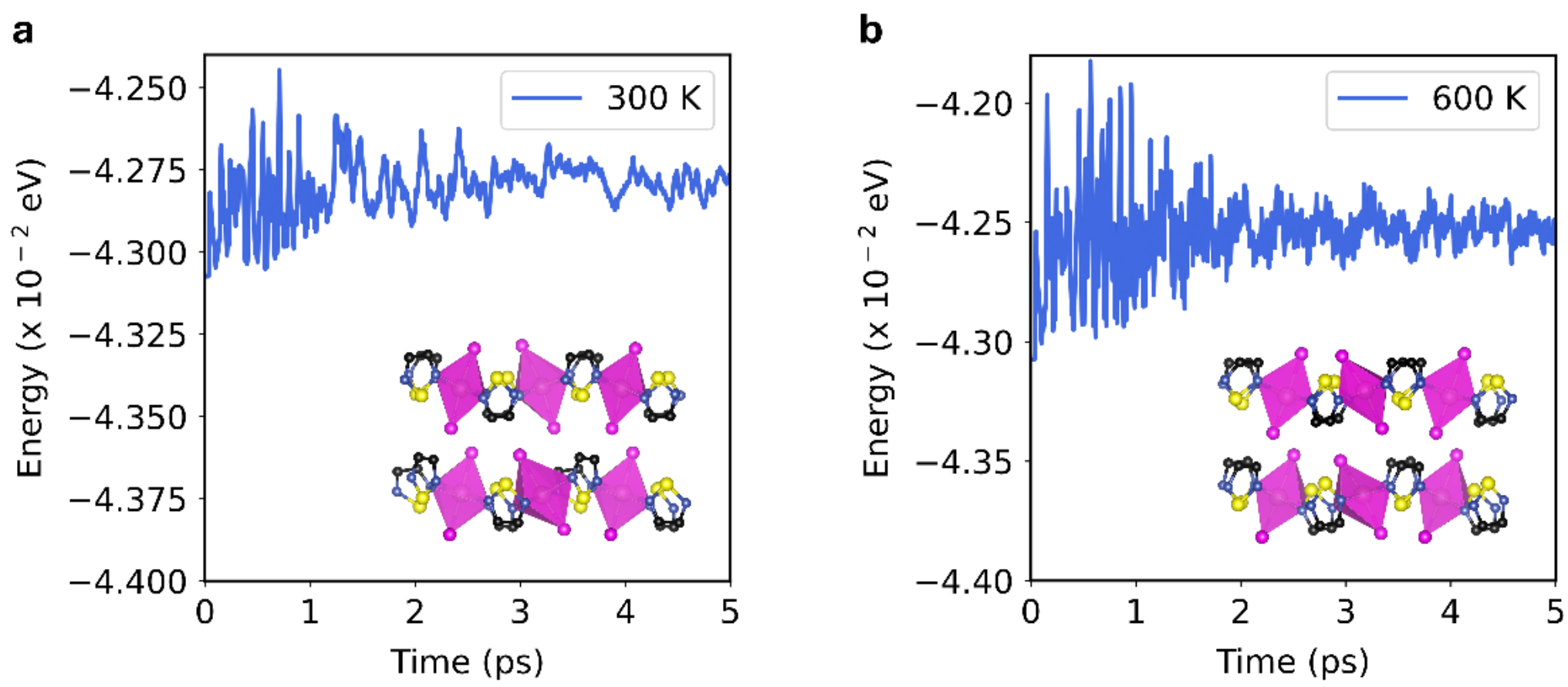

Figure S6. *Ab initio* molecular dynamics simulations in $MnCl_2(tdz)_2$ at **a** 300 K and **b** 600 K. Inset figures represent the last structure of the simulation.

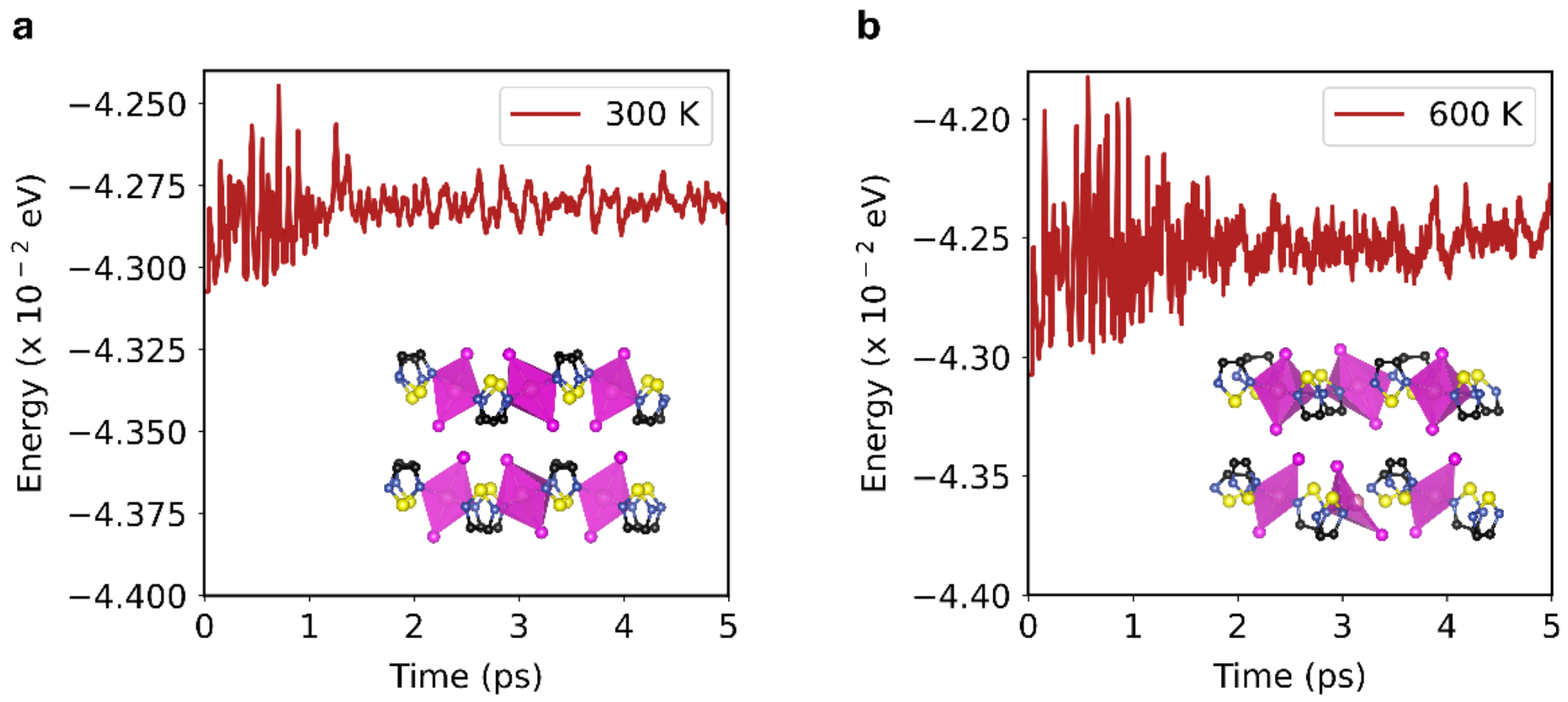

Figure S7. *Ab initio* molecular dynamics simulations in $MnBr_2(tdz)_2$ at **a** 300 K and **b** 600 K. Inset figures represent the last structure of the simulation.

Table S1. Calculated numerical error in $\Delta E_{AFM2-AFM3}$ MnBr2(tdz)2 as a function of representative computational parameters.

		**$\Delta E_{AFM2-AFM3}$ (meV/Mn atom)**
**k point mesh**	3 × 3 × 2	1.0227
	4 × 4 × 2	1.0227
	5 × 5 × 3	1.023
	6 × 6 × 3	1.023
**ENCUT (eV)**	450	1.0178
	500	1.023
	550	1.0185

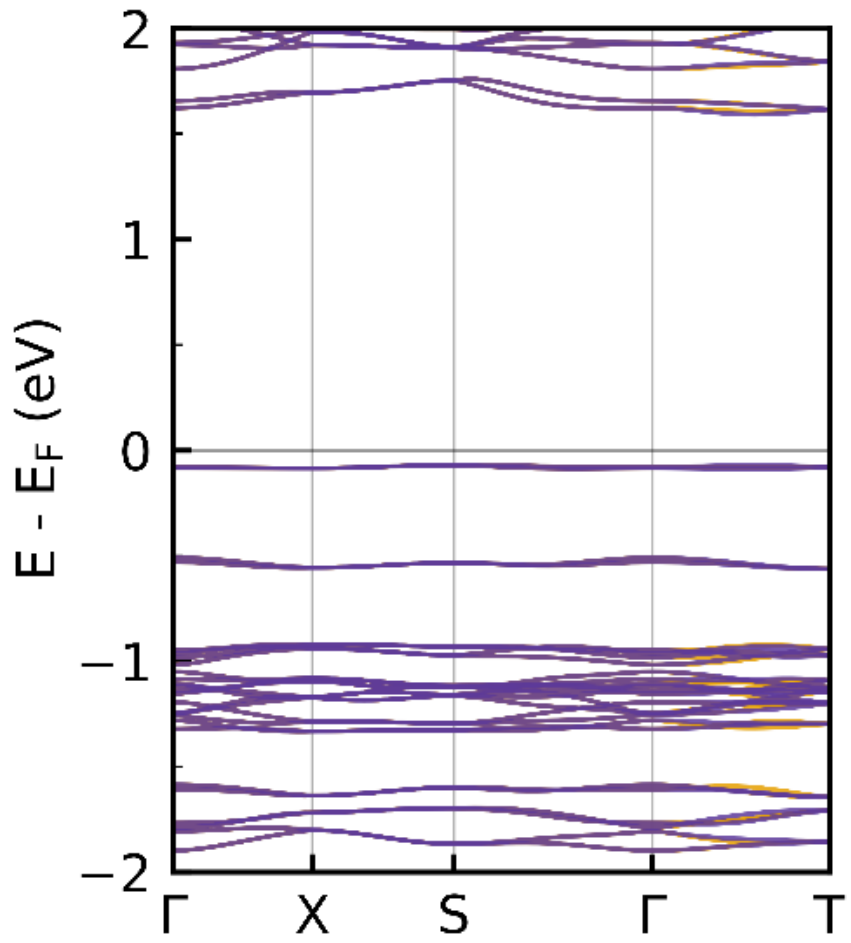

Figure S8. Electronic band structure of $MnBr_2(tdz)_2$ (U_{eff} = 3 eV).

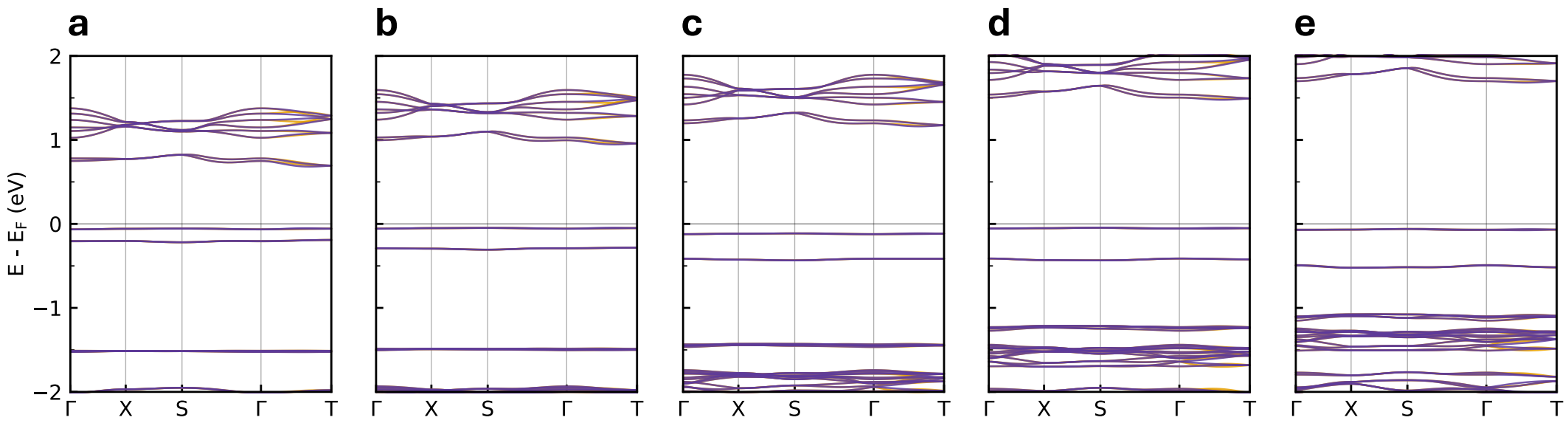

Figure S9. Calculated electronic band structure of $MnCl_2(tdz)_2$ with on-site U corrections of **a** 0, **b** 1, **c** 2, **d** 3 and **e** 4 eV.

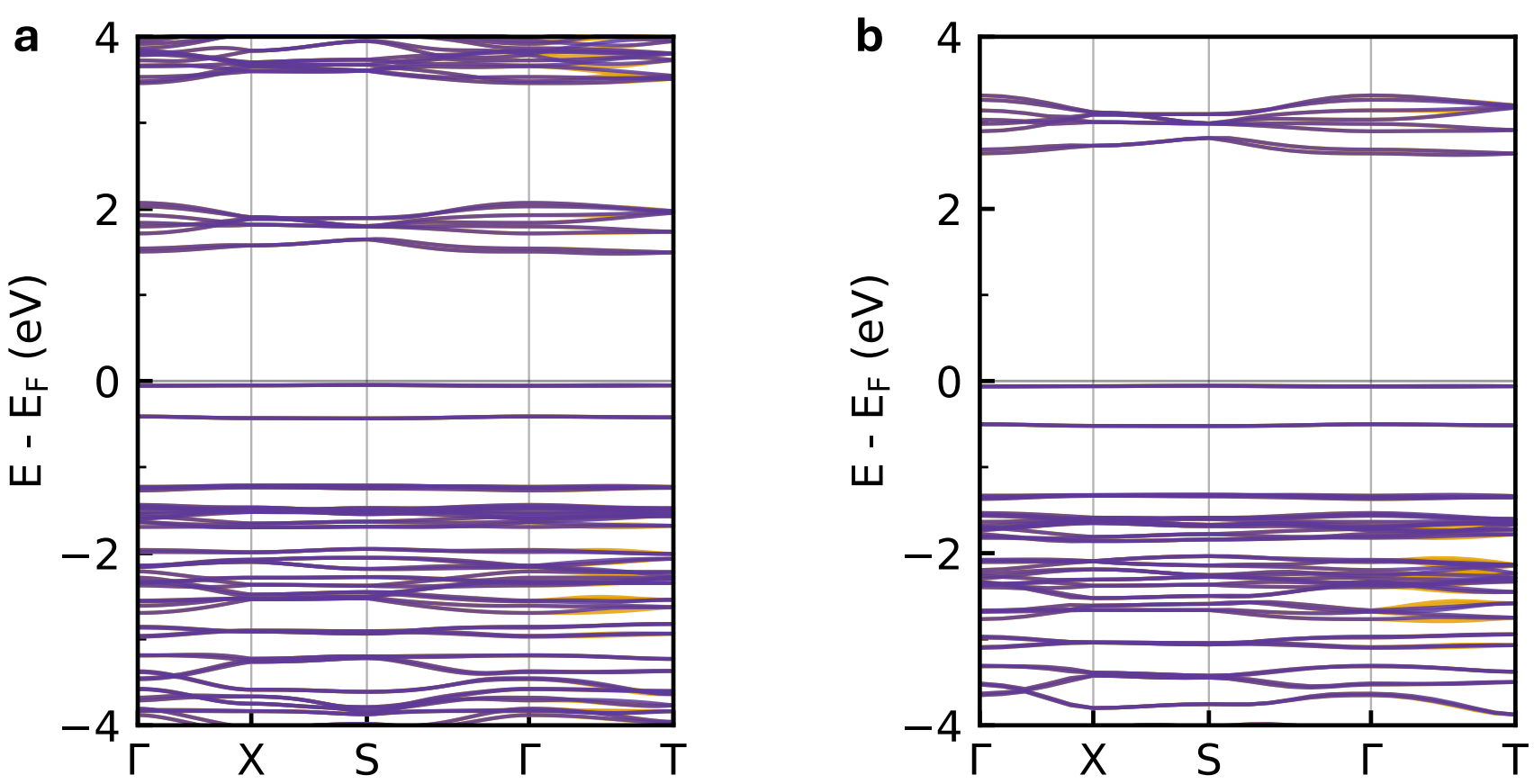

Figure S10. Calculated electronic band structure using **a** PBE+U (U_{eff} = 3 eV) and **b** HSE06 functionals in $MnCl_2(tdz)_2$.

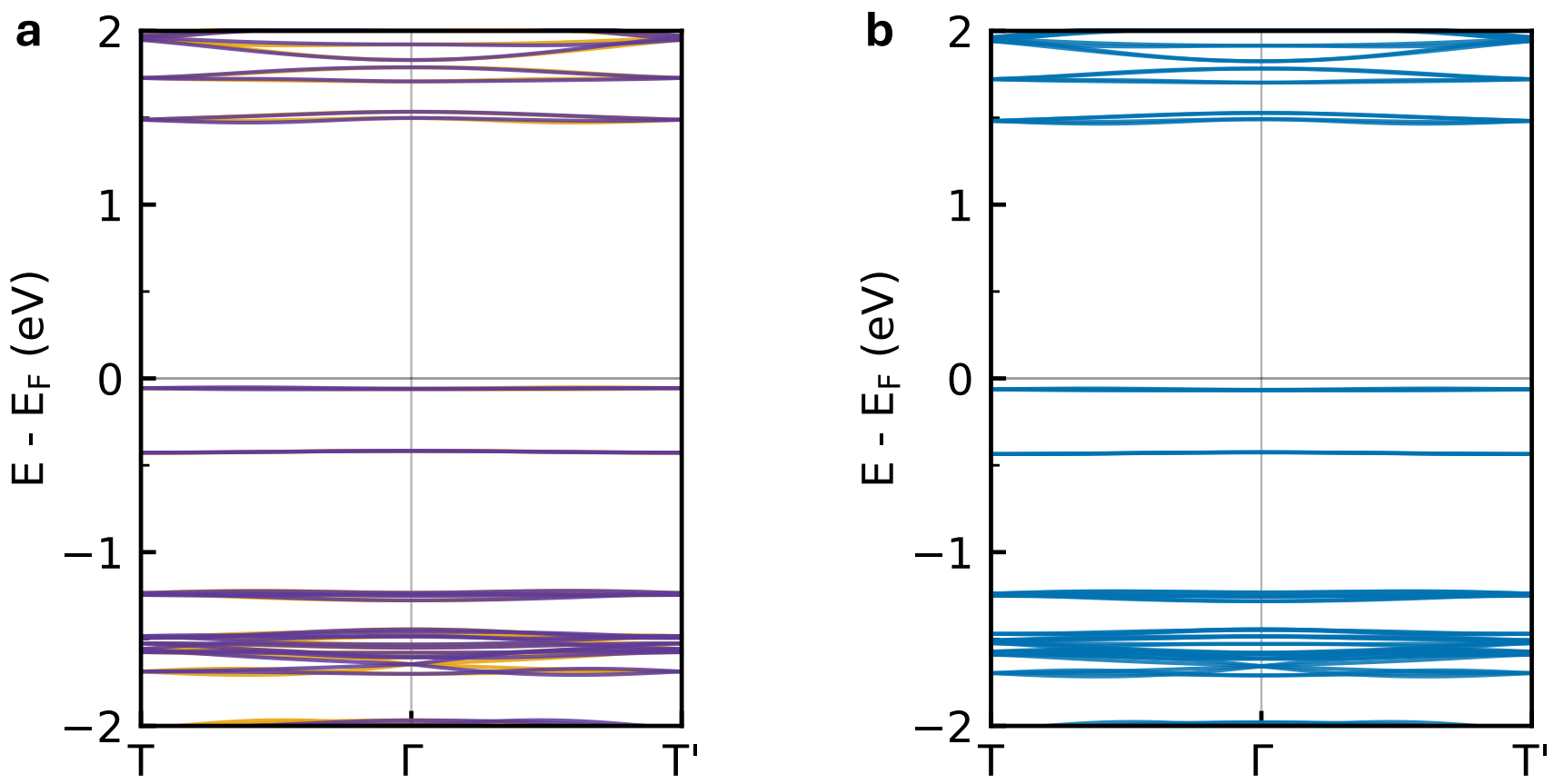

Figure S11. Calculated electronic band structure using PBE+U (U_{eff} = 3 eV) **a** without SOC and **b** with SOC in $MnCl_2(tdz)_2$.

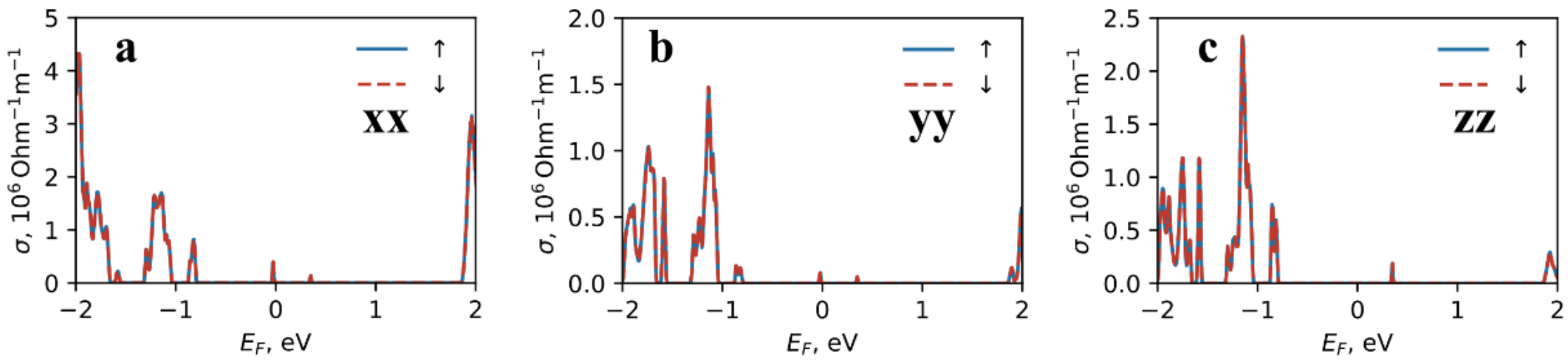

Figure S12. Diagonal terms of the conductivity tensor calculated via BoltzWann in $MnCl_2(tdz)_2$ at P = 0 GPa and T = 20 K. Color code: blue (red) represents spin up (down).

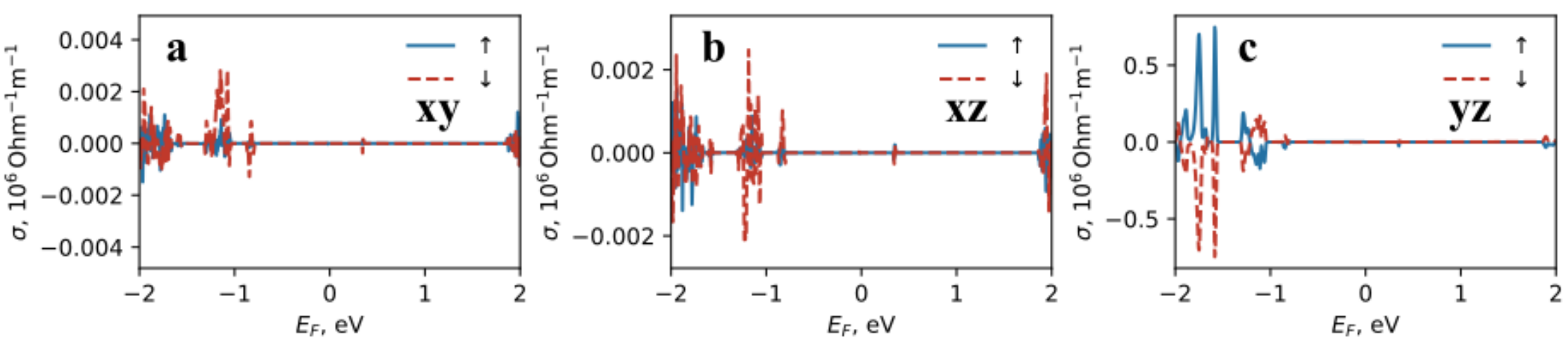

Figure S13. Off-diagonal terms of the conductivity tensor calculated via BoltzWann in $MnCl_2(tdz)_2$ P = 0 GPa and T = 20 K. Color code: blue (red) represents spin up (down).

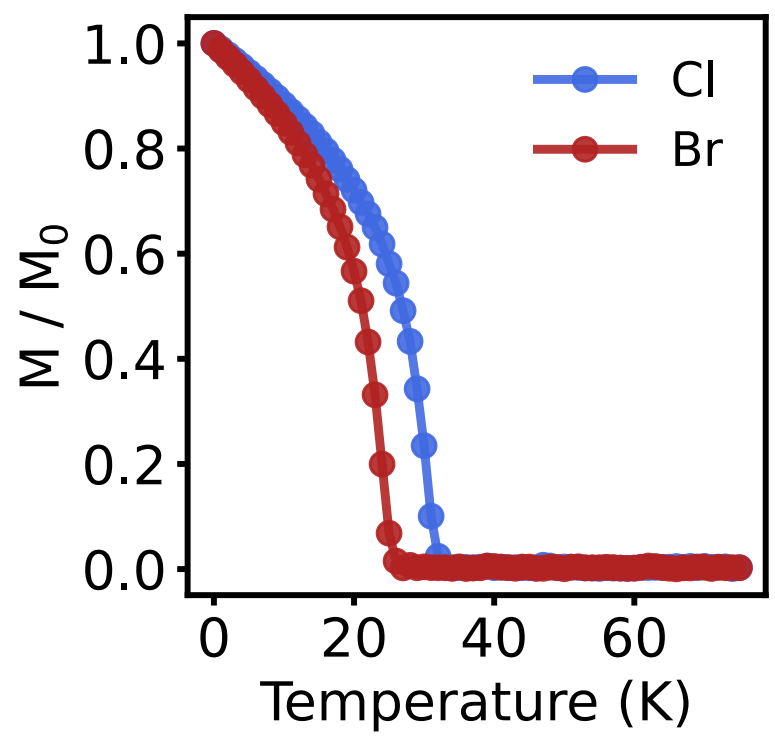

Figure S14. Magnetization as a function of temperature simulated via atomistic spin simulations in $MnX_2(tdz)_2$.

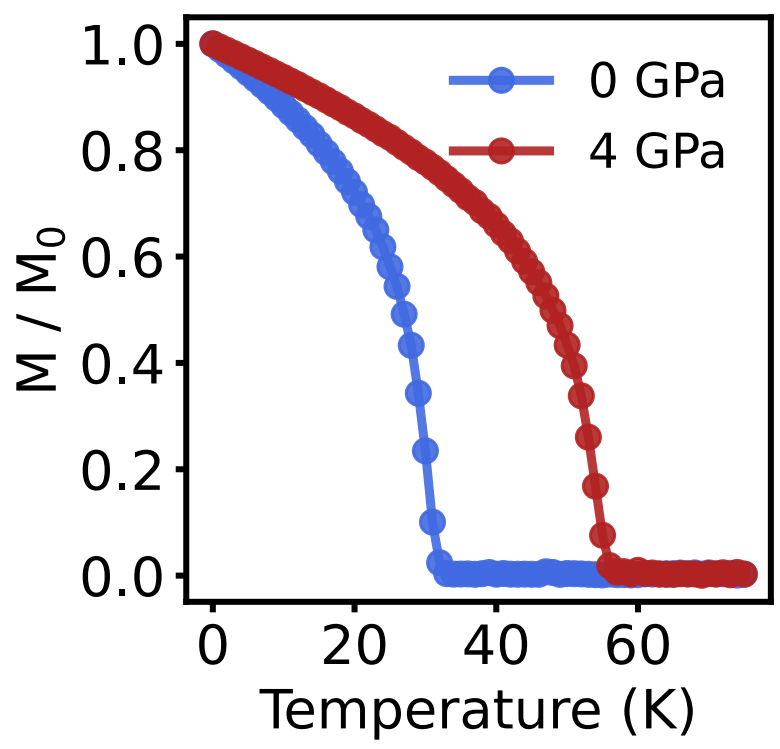

Figure S15. Magnetization as a function of temperature simulated via atomistic spin simulations in $MnCl_2(tdz)_2$ at different pressures.

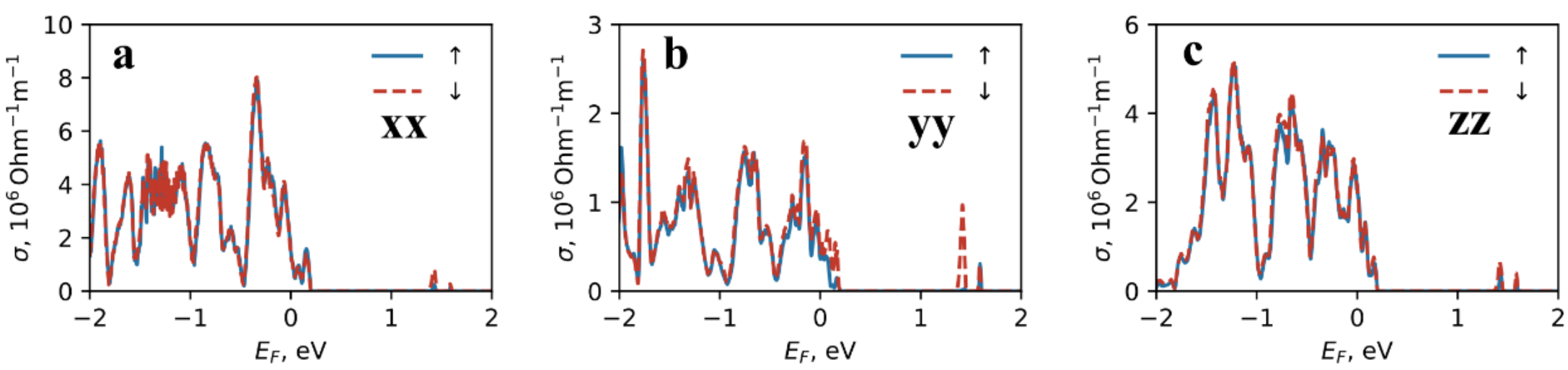

Figure S16. Diagonal terms of the conductivity tensor calculated via BoltzWann in $MnCl_2(tdz)_2$ at P = 4 GPa and T = 20 K. Color code: blue (red) represents spin up (down).

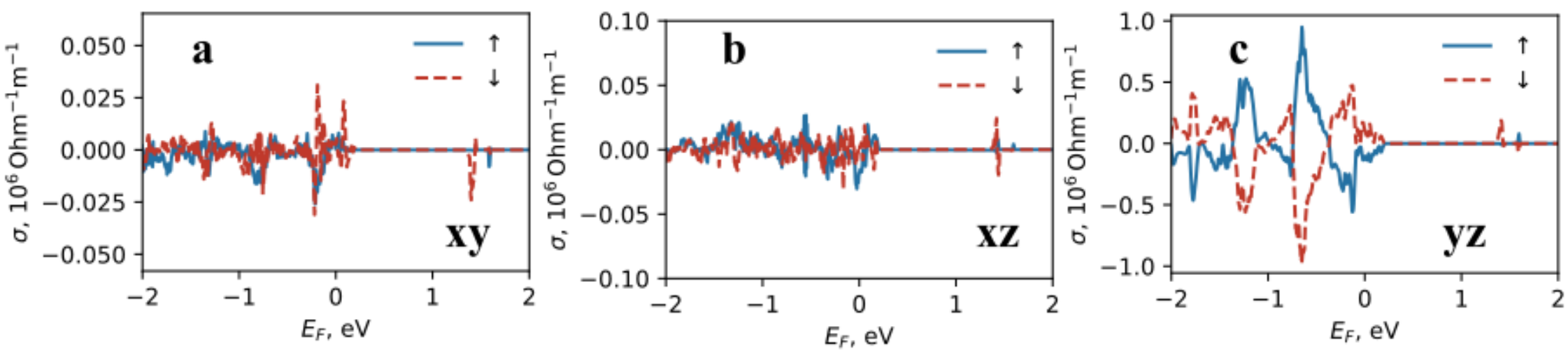

Figure S17. Off-diagonal terms of the conductivity tensor calculated via BoltzWann in $MnCl_2(tdz)_2$ at P = 4 GPa and T = 20 K. Color code: blue (red) represents spin up (down).

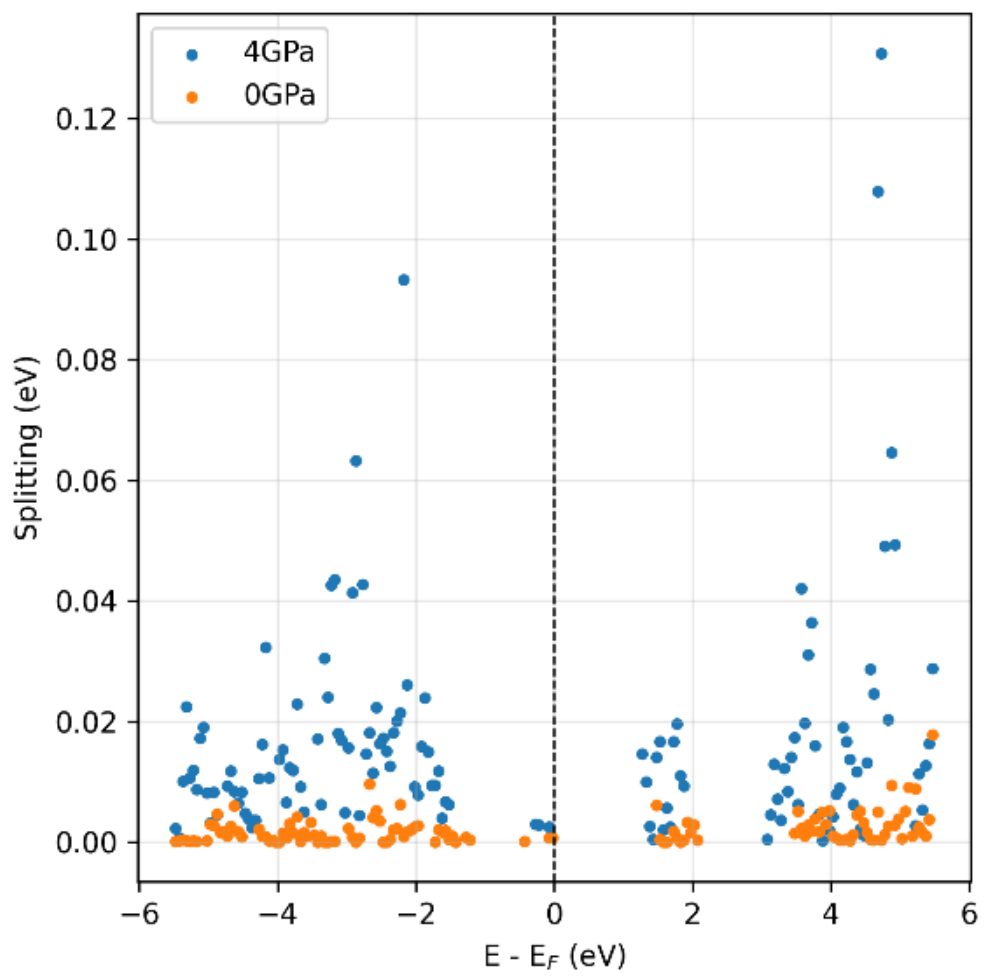

Figure S18. Calculated AM spin splitting in $MnCl_2(tdz)_2$ at P = 0 (orange) and 4 (blue) GPa.

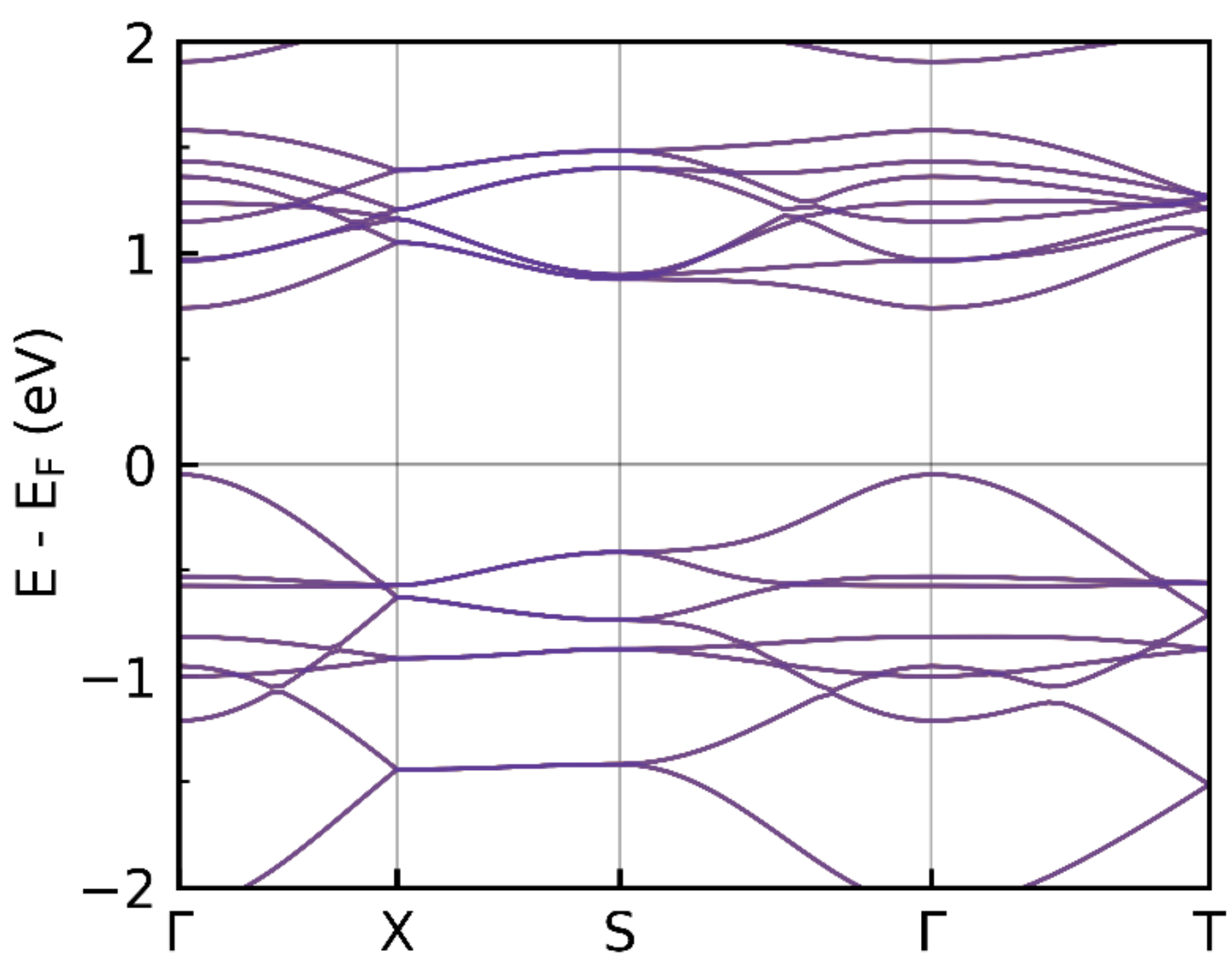

Figure 19. Electronic band structure of $MnCl_2(tdz)_2$ at P = 5 GPa.

References

(1) Valet, T.; Fert, A. Theory of the Perpendicular Magnetoresistance in Magnetic Multilayers. *Phys. Rev. B* **1993**, *48* (10), 7099–7113.

(2) Xie, L. S.; Skorupskii, G.; Dincă, M. Electrically Conductive Metal–Organic Frameworks. *Chem. Rev.* **2020**, *120* (16), 8536–8580.

(3) Wang, Y.; Zhou, Z.; Wen, T.; Zhou, Y.; Li, N.; Han, F.; Xiao, Y.; Chow, P.; Sun, J.; Pravica, M.; Cornelius, A. L.; Yang, W.; Zhao, Y. Pressure-Driven Cooperative Spin-Crossover, Large-Volume Collapse, and Semiconductor-to-Metal Transition in Manganese(II) Honeycomb Lattices. *J. Am. Chem. Soc.* **2016**, *138* (48), 15751–15757.
